# Breast cancer risk in female survivors of Hodgkin lymphoma after exposure to non-pegylated liposomal doxorubicin at young adult and adult age: the first real-life series from Southern Italy cancer centers

**DOI:** 10.1007/s00277-026-07173-z

**Published:** 2026-07-16

**Authors:** Marco Picardi, Annamaria Vincenzi, Claudia Giordano, Novella Pugliese, Elena Vigliar, Giancarlo Troncone, Rosaria Cappiello, Massimo Mascolo, Ciro Mainolfi, Rosa Fonti, Silvana Del Vecchio, Marianna Carchia, Alessia Salemme, Vincenzo Damiano, Roberto Bianco, Marcello Persico, Fabio Trastulli, Mario Annunziata, Rossella Iula, Catello Califano, Fabrizio Pane

**Affiliations:** 1 Department of Clinical Medicine and Surgery, Federico II University Medical School, Via Sergio Pansini, 5, Naples, Naples, 80131 Italy; 2 Department of Public Health, Federico II University Medical School Naples, Via Sergio Pansini, 5, Naples, Naples, 80131 Italy; 3 Department of Advanced Biomedical Sciences, Federico II University Medical School, Via Sergio Pansini, 5, Naples, 80131 Italy; 4 Department of General Surgery, Endocrinology, Orthopaedics, and Rehabilitations, Federico II University Medical School Naples, Via Sergio Pansini 5, Naples, 80131 Italy; 5 Hematology Unit, Antonio Cardarelli Hospital of national importance, Naples, Italy; 6 Hematology Unit, Hospital Andrea Tortora, Via Alcide De Gasperi, 59, Pagani, 84016 Italy

**Keywords:** Hodgkin lymphoma, Non-pegylated liposomal doxorubicin, MBVD, Radiotherapy, Breast cancer risk, YA&A

## Abstract

Studies in female Hodgkin lymphoma (HL) survivors have shown that doxorubicin hydrochloride exposure at young adult and adult (YA&A) age is associated with an increased risk of breast cancer (BC) regardless of chest-radiotherapy (c-RT). Although non-pegylated liposomal doxorubicin (NPLD)-based regimens have shown efficacy and safety in the treatment of lymphoma, the association between NPLD exposure and BC risk has not been examined in HL survivors. We assessed standardized incidence ratio (SIR) and absolute excess risk (AER) of BC in a cohort of 100 female ≥ 5-year HL survivors treated between 18 and 60 years of age with NPLD-based regimen in tertiary hospitals in southern Italy between 2009 and 2020. The median cumulative liposomal doxorubicin dose was 290 mg/m²; c-RT was administered to 20% of patients. After a median follow-up of 10 years (interquartile range, 8–11 years), no women had developed BC. Compared with the Italian general population, the SIR was 0.00 (95% confidence interval, 0.00–4.29); the standardized ratio was 86.5 per 100,000 person per year according to the AIRTUM (Associazione Italiana Registri Tumori) Working Group data for women aged between 18 and 60 years. In our series, the AER was − 8.87 per 10,000 person per year. Based on data derived from the pooled summary rate of literature, HL survivors treated with doxorubicin hydrochloride-including regimens at YA&A age had an incidence rate of BC of 1.6% at 10-year median follow-up. These data, if validated in largest datasets with longer follow-up, suggest that treatment with NPLD could be not associated with increased BC risk in HL survivors, unlike conventional doxorubicin. Our findings provide a rationale for evaluating NPLD-based frontline therapy in prospective studies of YA&A patients with newly diagnosed HL.

**Supplementary Information:**

The online version contains supplementary material available at https://doi.org/10.1007/s00277-026-07173-z.

## Key points

This study evaluated whether exposure to non-pegylated liposomal doxorubicin (NPLD) during young adult and adult (18–60 years) age is associated with subsequent breast cancer (BC) risk in female ≥5-year survivors of Hodgkin lymphoma (HL). After a median follow-up of 10 years, no BC events were observed among 100 female HL survivors treated with frontline NPLD-based chemotherapy, most of whom received cumulative liposomal doxorubicin doses >200 mg/m². No BC events were observed irrespective of age at HL treatment, chest radiotherapy exposure, or gonadotoxic therapy. A review of published cohorts of HL survivors treated with conventional doxorubicin-based regimens during young adult and adult age showed cumulative BC incidences ranging from <1% to 5%, with a descriptive sample-size–weighted estimate of approximately 1.6% after about 10 years of follow-up. Although these findings require confirmation in larger cohorts with longer follow-up, they support further prospective evaluation of NPLD-based frontline regimens as a potential strategy to optimize the long-term safety profile of HL treatment while maintaining the efficacy of anthracycline-based therapy.

**Supplementary Information:**

The online version contains supplementary material available at https://doi.org/10.1007/s00277-026-07173-z.

## Introduction

Several studies have reported an increased risk of breast cancer (BC) among female Hodgkin lymphoma (HL) survivors treated with anthracycline-based chemotherapy, even in the absence of chest radiotherapy (c-RT) [1–3]. Among the agents included in the ABVD regimen (doxorubicin, bleomycin, vinblastine, and dacarbazine), cumulative exposure to doxorubicin hydrochloride, particularly at doses > 200 mg/m², has been associated with an increased BC risk and is thought to promote carcinogenesis through DNA damage and immune dysregulation [3–8]. Although anthracycline-based chemotherapy remains associated with excellent clinical outcomes in HL [9, 10], strategies aimed at reducing exposure to doxorubicin hydrochloride particularly in young adult and adult (YA&A) are desirable to mitigate the long-term risk of chemotherapy-related BC.

Advances in liposome drug delivery have led to the development of non-pegylated liposomal doxorubicin (NPLD), a stable, doxorubicin encapsulated in a non-pegylated liposomal membrane of phosphatidylcholine and cholesterol [11, 12]. Liposomal encapsulation modifies the pharmacokinetic profile of doxorubicin, enabling preferential accumulation within neoplastic tissue, as demonstrated in both preclinical models and human solid tumors¹³^,^¹⁴. In phase II trials evaluating frontline treatment strategies for HL, NPLD (Myocet™) was combined with bleomycin, vinblastine, and dacarbazine, replacing conventional doxorubicin and defining a novel MBVD regimen (consisted of 25 mg/m^2^ of NPLD plus standard bleomycin, vinblastine and dacarbazine) [13, 14]. Liposomal doxorubicin–based regimens demonstrated favourable antitumor activity, with efficacy comparable to historical ABVD outcomes and an improved toxicity profile¹⁵-¹⁸. However, to date, the association between NPLD exposure and BC risk has not yet been specifically investigated in HL survivors. Whether NPLD exposure is associated with BC risk remains unknown. Clarifying this association may have implications for treatment selection in newly diagnosed female HL patients.

In Italy, the national regulatory agency (AIFA) has approved the frontline use of NPLD-based regimens in lymphoma patients from several years, based on their favourable, safety and efficacy profiles [15–17]. In four tertiary referral centers in southern Italy, a treatment strategy incorporating liposomal doxorubicin has been routinely adopted for patients with previously untreated stage IIB–IV HL. This approach consisted of up-front administration of six cycles of MBVD followed by consolidative radiotherapy when clinically indicated, as previously described [13, 14, 18–22]. Here, we report the first real-world cohort of female HL survivors who were alive ≥ 5 years after frontline treatment with liposomal doxorubicin–based chemotherapy full course at YA&A age, with or without c-RT. The aim was to assess the BC risk in this population.

## Patients and methods

### Patient selection

Between November 1, 2009 and September 30, 2020, we retrospectively identified consecutive female aged 18–60 years with previously untreated, biopsy-proven Hodgkin lymphoma (HL) [23–25] and Ann Arbor stage IIB–IV who received frontline MBVD chemotherapy followed by consolidative radiotherapy when clinically indicated, as previously described [13, 14, 18–22]. Women who were alive at least 5 years after completion of frontline treatment were eligible for inclusion. All HL diagnoses were made according to the World Health Organization Classification for Lymphoid Neoplasms by expert hematopathologists applying the pathological criteria current at the time of diagnosis. We conducted a retrospective, multi-center study based on the medical records and/or local databases of the Hematology Unit of the Federico II University of Naples (Italy), Oncology Unit of the Federico II University of Naples (Italy), Hematology Unit of the Antonio Cardarelli Hospital of national importance of Naples (Italy) and Hematology Unit of the Andrea Tortora Hospital of Pagani (Italy). These four tertiary care centres shared similar institutional guidelines for HL management, regarding the upfront therapy, and diagnostic work-up during post-treatment surveillance, with specific imaging techniques to detect disease relapse as well as secondary malignancies.

Breast cancer surveillance was performed according to the institutional follow-up protocol adopted at the participating centers. All female HL survivors, irrespective of chest radiotherapy exposure, underwent routine clinical breast examination and biennial breast imaging with ultrasonography and/or mammography throughout follow-up. Additional imaging investigations, including breast magnetic resonance imaging, were performed when clinically indicated. Suspicious breast lesions were evaluated by imaging-guided core needle biopsy and/or surgical biopsy according to standard institutional procedures. This surveillance strategy reflected the institutional follow-up protocol adopted at the participating centers and was based on the concern that both radiotherapy and systemic treatment may contribute to long-term secondary malignancies [26–30]. All necessary approvals were obtained from the ethics committee (approval protocol number: 62/2025; Segreteria CET Campania 3; segreteria@comitatoeticocampania3.it) and ClinicalTrials.gov (number, NCT07120217). The study was undertaken in accordance with the Declaration of Helsinki. All patients provided written informed consent for personal data analysis for research purposes.

### Study design

A minimum follow-up of 5 years after completion of frontline treatment was required to ensure an adequate latency period for BC risk assessment. This threshold was chosen because previous studies have shown that the risk of secondary BC in HL survivors begins to increase approximately 5 years after treatment [1–3]. Accordingly, patients treated between November 1, 2009, when liposomal doxorubicin was introduced into routine clinical practice at the participating centers, and September 30, 2020, were eligible for inclusion. We describe a multicentric retrospective study evaluating BC risk in females who received frontline treatment with MBVD ± c-RT for stages II-B to IV HL at 18–60 years age, in tertiary oncology centers in Italy. The primary composite objective was to estimate the standardized incidence ratio (SIR) and the absolute excess risk (AER) of BC [31]. Cumulative BC incidence was not selected as the primary endpoint because the sample size was too small and the follow-up too short to provide reliable cumulative incidence estimates. The secondary objective was to review published evidence on BC risk following conventional doxorubicin-based frontline regimens in HL survivors treated during young adult and adult age, and to derive a descriptive sample-size–weighted estimate of BC incidence across the available studies [1–3, 6, 32, 33].

### Data collection

We collected data from medical records and/or local databases on consecutive patients with stage IIB-IV HL (supplemental methods: Data Collection) who were treated with frontline MBVD at YA&A age (supplemental methods: MBVD Treatment Plan). Additional therapeutic details were also retrieved, including radiotherapy exposure (fields and delivered dose; Supplemental Methods: Irradiation), administration of GnRH analogues (number of decapeptyl cycles), and vital status with date of death. Cumulative doses of liposomal doxorubicin were calculated based on the number of cycles of chemotherapy administered and categorized as follows: 1-100 mg/m^2^ (two cycles of MBVD), > 100 mg/m^2^ to 200 mg/m^2^ (four cycles of MBVD), > 200 mg/m^2^ (six cycles of MBVD). Data on subsequent secondary malignancies were collected directly from medical records.

### Inclusion criteria

Eligibility criteria included the followings: histological diagnosis of HL; women ≥ 18 and ≤ 60 years of age at HL diagnosis; frontline treatment of HL by using MBVD ± c-RT; survival in first complete hematologic remission for at least 5 years from the date of completion of treatment; and regular post-treatment follow-up according to routine imaging diagnostic tools [26–30]. Survivors with missing data on treatment or follow-up variables were excluded (complete case analysis).

### Identification and confirmation of subsequent breast cancer

BC events including both invasive carcinomas and ductal carcinomas in situ, were evaluated for the final analysis. Any subsequent malignancy occurring after HL other than BC was excluded from all analyses.

### Statistical analysis

Comparison with the expected number of BC cases was based on age- and calendar year-specific BC incidence rates for the general female population in Italy, obtained from the AIRTUM (Associazione Italiana Registri Tumori) Working Group data [31]. The SIR and AER, per 10,000 person-years, together with their 95% confidence intervals (CIs), were calculated using standard methods (detailed procedures are provided in the Supplemental data). HL survivors were considered at risk for BC beginning 5 years after their completion of frontline treatment until the earliest occurrence of BC diagnosis, secondary malignant neoplasm (SMN) other than BC, HL recurrence, death, or last known follow-up. Individuals who developed HL recurrence or an SMN other than BC were censored at the date of the event, as the analysis especially focused on BC risk among ≥ 5 years-survivors after liposomal doxorubicin-based frontline therapy. Thus, for all included patients time at risk began 5 years after completion of frontline treatment and ended at the date of BC diagnosis, death, or the censoring date which ever occurred first. All statistical analyses were performed using R software (version 4.3.1). All tests were two-sided, and a p-value < 0.05 was considered statistically significant.

## Results

### Characteristics of HL survivors

A total of 100 female ≥ 5-year survivors of HL who had received frontline liposomal doxorubicin-based regimen for advanced-stage HL were included in the final analysis (Table 1). The median age at first treatment was 32.6 years (IQR range, 18–59 years). Most survivors (90%) were treated with six cycles of MBVD, while the remaining 10% received two to five cycles of MBVD for acute toxicity reasons. Cumulative liposomal doxorubicin doses were categorized as follows: 201–300 mg/m² (median, 290 mg/m²) in 90 patients, 101–200 mg/m² (median, 150 mg/m²) in 6 patients, and 1-100 mg/m² (median, 90 mg/m²) in 4 patients. c-RT was administered to 20 patients; among the remaining 80 patients, 10 received irradiation not including mediastinal fields (abdominal and/or pelvic lymph node fields for 30 Gy), whereas 70 underwent follow-up without any additional treatments. A GnRH analogue was administered in 60 women.

**Table 1:** Patient characteristics at Hodgkin lymphoma diagnosis

Characteristics	Total (*N* = 100)	(%)
Median age, (range) years	32.6 (18–59)	–
Ann Arbor Stage		
Stage II	22	22
Stage III	55	55
Stage IV	23	23
B symptoms	33	33
MBVD cycles administered		
2 cycles	4	4
4 cycles	6	6
6 cycles	90	90
Cumulative liposomal doxorubicin dose		
1–100 mg/m² (median 90 mg/m²)	4	4
101–200 mg/m² (median 150 mg/m²)	6	6
201–300 mg/m² (median 290 mg/m²)	90	90
Radiotherapy (RT)		
c-RT	20	20
Other sites of RT	10	10
No RT after chemotherapy	70	70
GnRH analogue administration		
Received GnRH analogue	30	30

Values are n (%) unless otherwise specified.*HL* Hodgkin lymphoma, *MBVD* non-pegylated liposomal doxorubicin, bleomycin, vinblastine, dacarbazine, *RT* radiotherapy, *c*-*RT* chest radiotherapy including mediastinal fields, *GnRH* gonadotropin-releasing hormone.Ann Arbor staging: stage I defined as involvement of a single lymph nodal site or a single extralymphatic organ or site; stage II: involvement of two or more lymph node sites on the same side of the diaphragm or localized involvement of an extralymphatic organ or site; stage III, defined as multiple lymph node sites on both sides of the diaphragm; stage IV, defined as multiple extra-nodal sites or lymph nodes and extra-nodal disease; B symptoms: fever, weight loss > 10% in the last 6 months, nocturnal sweat

### SIR and AER rates

After a median follow-up of 10 years (IQR range, 8–11 years), no BC cases were observed in our cohort. According to AIRTUM Working Group data, the age-specific BC incidence rate in the Italian general population aged 18–60 years is 86.5 per 100,000 person-years (Supplementary Statistical Analysis). Given that our cohort contributed approximately 1000 person-years of follow-up, the expected number of BC cases was 0.86. The SIR was 0.00 (95% CI, 0.00–4.29), calculated using exact Poisson limits for zero-event data. The AER was − 8.7 per 10,000 person-years. Thus, no excess BC incidence was observed among HL survivors treated with liposomal doxorubicin-containing frontline regimens during the available follow-up period.

### Breast cancer occurrence in predefined clinical subgroups

The absence of BC events was consistent across all predefined clinical subgroups (Supplementary Table 1). In particular, no BC cases were observed among patients receiving cumulative NPLD doses > 200 mg/m² (*n* = 90), women treated before 30 years of age (*n* = 35), patients exposed to chest radiotherapy (*n* = 20), or those receiving pelvic irradiation (*n* = 5). Similar findings were observed among women treated at 30–40 years (*n* = 43) and > 40 years of age (*n* = 22). Across these subgroups, the median follow-up was approximately 10 years, providing a comparable observation period for descriptive analyses.

## Discussion

**Fig. 1:**
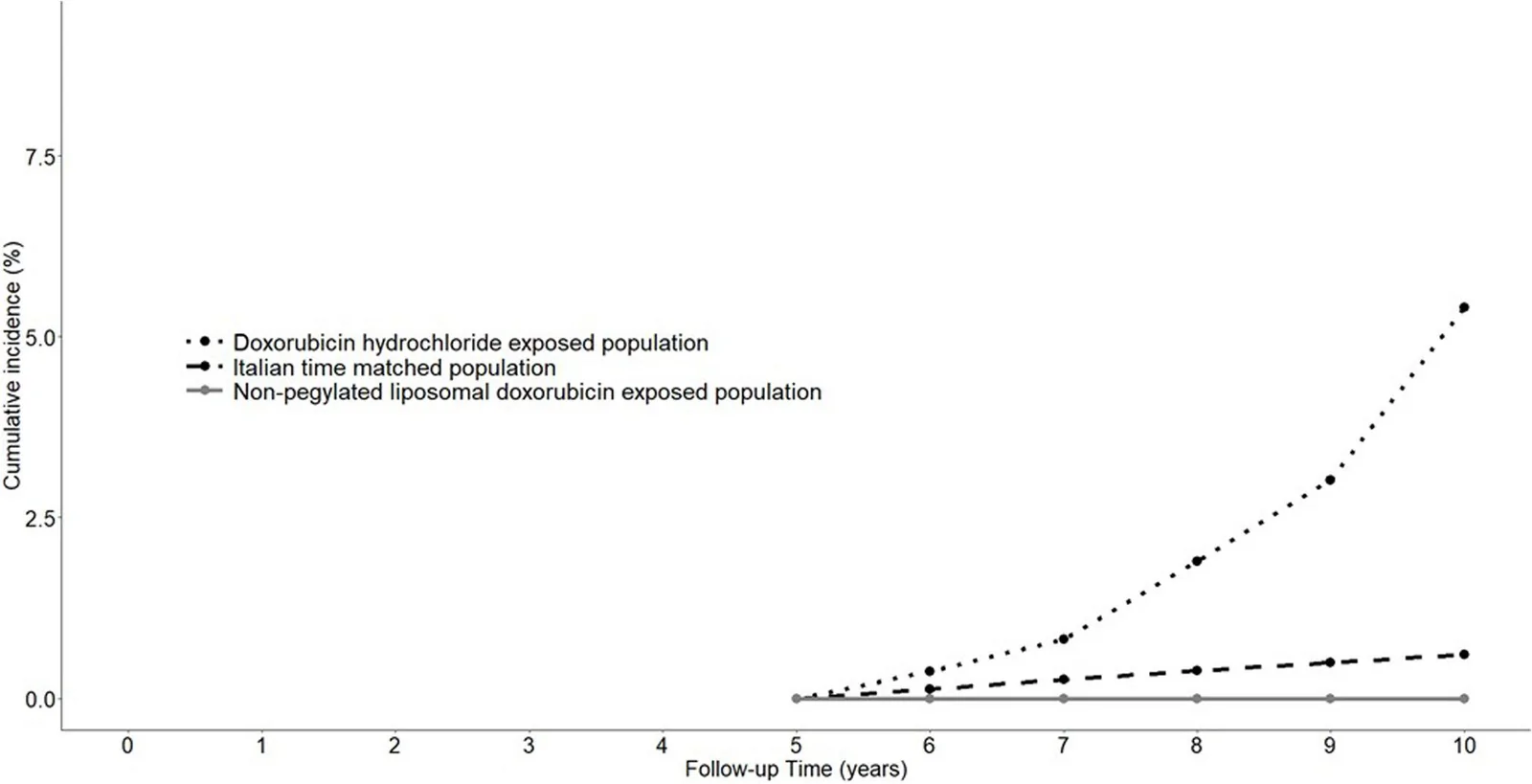
Cumulative incidence of BC (invasive BC and DCIS) in female ≥5-year HL survivors. Solid line represents the cumulative incidence of BC following non-pegylated liposomal doxorubicin-based treatment in our cohort. Dashed line represents the expected incidence of BC in the Italy general population matched by age and sex, stratified by the same periods. Dotted line represents the cumulative incidence curve at 10 year of BC following doxorubicin hydrochloride-based treatment derived from the published study by Laddaga et al. [32], which included Southern Italy female HL survivors, as explicitly reported in Table 2. BC, breast cancer; DCIS, ductal carcinoma in situ; HL, Hodgkin lymphoma

To our knowledge, this is the first study to date specifically evaluating the impact of liposomal doxorubicin, as part of the frontline therapy of HL at YA&A age, on BC risk in female ≥ 5-year survivors, thereby avoiding exposure to conventional doxorubicin. Our real-life study includes a series of advanced-stage HL cases homogenously treated with an intensive chemotherapy based on six cycles of MBVD alone for most patients, in absence of residual nodal masses (RNMs). The presence of RNMs in thorax sites occurred in 20% of patients; these cases underwent chest irradiation for 30 Gy. Most female survivors were exposed to a cumulative dose of liposomal doxorubicin of > 200 mg/m^2^. The principal finding of this study is that no BC events were observed during the available follow-up among female HL survivors treated with frontline NPLD-containing regimens. To contextualize our findings, we reviewed published cumulative BC incidence estimates from cohorts of HL survivors treated with conventional anthracycline-based frontline regimens. From a wide update review of the literature focusing on BC risk in HL women treated upfront with doxorubicin hydrochloride (or equivalents)-based regimens at YA&A age and who were ≥ 5-year survivors, we identified three original articles and conference abstract (Neppelenbroek et al. on Dutch HL survivars, Ehrhardt et al. on St Jude Children’s Research Hospital on North America HL survivars, Laddaga et al. on southern Italy HL survivars, and Machlab et al. on Canadian HL survivars). In these four cohorts, the median age at HL diagnosis was 26 years (range, 10–60 years) and we investigated baseline clinical characteristics, i.e., disease stage, number of chemotherapy courses administered, thorax irradiation rate and surveillance diagnostic work-up. Table 2 summarizes BC risk estimates reported in these studies. The four identified studies included a total of 3289 women treated with conventional doxorubicin or conventional doxorubicin-equivalent-containing chemotherapy, with reported cumulative BC incidences ranging from < 1% to 5% over approximately 10 years of follow-up. Across these reports, the 10-year cumulative incidence of BC individual estimates was of < 1.0% (Ehrhardt et al., St Jude Lifetime Cohort Study at St Jude Children’s Research Hospital [Memphis, USA]), 1.5% (Neppelenbroek et al., Dutch HL survivors), 1.71% (Machlab et al., Canadian HL survivors), and 5% (Laddaga et al., Southern Italy HL survivors). A descriptive sample-size–weighted estimate across studies was approximately 1.6%. We acknowledge, however, that the comparison with published conventional anthracycline cohorts is approximate, as it is based on indirect extrapolations by the authors using the data reported in each study rather than individual patient-level comparisons. These descriptive comparisons cannot be translated into hazard ratios because no BC events were observed in the NPLD cohort. Therefore, time-to-event modelling was not considered appropriate, and comparisons relied on descriptive incidence estimates. Although limited by indirect comparisons and heterogeneous follow-up across studies, no BC events were observed in the NPLD cohort during the available follow-up, whereas published conventional doxorubicin-based series reported measurable BC incidences in HL survivors treated at YA&A age. Although no direct statistical comparison was possible, published cohorts of female HL survivors treated with conventional doxorubicin-based frontline regimens consistently reported measurable BC incidences over long-term follow-up. Reported cumulative BC incidence ranged from < 1% to 5% (Fig.  1), with a descriptive sample-size–weighted estimate of approximately 1.6% [1, 2, 32, 33]. Previous studies have also shown that cumulative conventional doxorubicin doses > 200 mg/m² are associated with increased BC risk, which further increases by approximately 18% for every additional 100 mg/m² administered [1, 2, 32, 33]. These observations are biologically plausible. Conventional doxorubicin induces DNA damage through double-strand breaks and chromatin disruption via histone eviction, mechanisms that may contribute to genomic instability and late carcinogenesis [4–8]. By contrast, NPLD displays a distinct pharmacokinetic profile, with reduced distribution to healthy tissues while maintaining antitumor activity, thereby potentially limiting off-target toxicity [11–14, 34–37]. Although these pharmacological differences may explain the absence of observed BC events in our cohort, the present findings should be interpreted cautiously given the limited sample size and follow-up.

**Table 2:** Breast cancer risk in women ≥ 5-year HL survivors receiving upfront liposomal doxorubicin versus conventional anthracyclines-based regimens at young adult and adult age

Study	Anthracycline-based therapy ± irradiation	*N*. of patients analyzed	Median age (range), years	Follow-up window	Cumulative BC incidence*
Current study (Picardi et al., Southern Italy HL survivors)	Liposomal doxorubicin (MBVD) c-RT (20%)	100	32.6 (18–59)	10 years	0%
Neppelenbroek et al., JCO 2024 (Dutch HL survivors)	Adriamycin (57%) Epirubicin (10%) Equivalents (33%) c-RT (47%)	1964	27.8 (15–50)	10 years	1.5%
Machlab et al., Blood 2024 (Canadian HL survivors)	Adriamycin c-RT (50%)	1089	28.5 (11–60)	10 years	1.71%
Laddaga et al., Cancers 2024 (Southern Italy HL survivors)	Adriamycin (ABVD) c-RT (50%)	62	25 (12–36)	10 years	5%
Ehrhardt et al., JCO 2019 (SJLIFE, HL survivors)	Adriamycin c-RT (29%)	174	15 (10–20)	10 years	< 1%

Abbreviations: *BC* breast cancer, *HL* Hodgkin lymphoma, *MBVD* myocet (non-pegylated liposomal doxorubicin), bleomycin, vinblastine, and dacarbazine, *ABVD* doxorubicin, bleomycin, vinblastine, and dacarbazine, *SJLIFE* St Jude Lifetime Cohort Study at St Jude Children’s Research Hospital (Memphis, USA), *c*-*RT* chest radiotherapy

Equivalent, it included conventional anthracycline drugs

*Estimated cumulative incidence derived from published cumulative incidence curves when numerical values at 10 years were not explicitly reported. For the Dutch cohort [1], the estimate reflects a pooled visual approximation across treatment periods with overlapping curves and no significant heterogeneity. For SJLIFE Hodgkin lymphoma survivors [2], early breast cancer incidence was very low, visually < 1%. For the Laddaga et al. southern Italy cohort [32], the cumulative incidence of BC was 6.5% at 15 years. For the Canadian cohort [33]

There is a clear need to maintain the high efficacy of the front-line therapy of HL with anthracycline-based regimens while minimizing treatment-related adverse events, underscoring the importance of developing truly efficacy- and safety-oriented therapeutical strategies. Several contemporary frontline regimens for advanced-stage HL, including BV-AVD (ECHELON-1), BrECADD (HD21), and nivolumab-AVD (SWOG S1826), continue to incorporate conventional doxorubicin as the anthracycline backbone [38–41]. Although these strategies have improved progression-free survival and reduced the use of consolidative radiotherapy, cumulative doxorubicin hydrochloride exposure remains substantial, ranging from approximately 160 to 300 mg/m². Moreover, follow-up remains insufficient to assess the long-term incidence of secondary malignancies, including BC. Whether replacing conventional doxorubicin with NPLD could preserve efficacy while improving long-term safety deserves prospective evaluation. Longer observation will be crucial, and these studies are expected to provide more definitive insights into the long-term safety of these treatment combinations in the coming years.

Our study has some limitations. First, the study has a short follow-up without data on long-term and/or very late anthracycline-induced toxicity. The association of anthracycline with BC risk has been reported to emerge predominantly after ≥ 15–20 years from HL treatment. Additional major limitations include the small sample size of analyzed patients. Both these limitations could explain that despite 20% of the population receiving chest irradiation (strong treatment-associated risk factor for subsequent BC) no BC occurred in our study. The analysis could be underpowered to detect BC risk increase, due to insufficient follow-up and sample size. Therefore, our results should be considered hypothesis-generating rather than definitive, i.e., practice-informing, and more comprehensive studies are needed.

In conclusion, our findings, if confirmed in larger cohorts with longer follow-up, suggest that the choice of anthracycline formulation may represent an additional factor to consider when balancing long-term therapy-related risks with conventional doxorubicin. At the same time, anthracycline-based chemotherapy platforms remain the cornerstone of front-line treatment for HL, given their consistently robust efficacy. In this context, frontline strategies incorporating non-pegylated liposomal doxorubicin, with the aim of reducing cumulative exposure to conventional doxorubicin, warrant further investigation and may be clinically relevant in optimizing the long-term safety profile of HL therapy [14].

## Supplementary Information

Below is the link to the electronic supplementary material.

Supplementary Material 1

## Data Availability

The data supporting the findings of this study are not publicly available because they contain information that could compromise the privacy of research participants. De-identified data may be made available from the corresponding author upon reasonable request and subject to approval by the relevant ethics committee and participating institutions.
