# Supplementary material for: Breast cancer risk in female survivors of Hodgkin lymphoma after exposure to non-pegylated liposomal doxorubicin at young adult and adult age: the first real-life series from Southern Italy cancer centers
Source: Ann Hematol. 2026 Jul 16;105(11):453. doi: 10.1007/s00277-026-07173-z (PMC13658420; doi:10.1007/s00277-026-07173-z)
Supplement: Supplementary file 1 [file 277_2026_7173_MOESM1_ESM.odt]

Supplemental file
Supplemental methods
Data Collection
Detailed information regarded demographic data, previous history of malignancy, Hodgkin lymphoma (HL) diagnosis, disease characteristics, Eastern Cooperative Oncology Group Performance Status (ECOG PS), laboratory values, primary treatment (chemotherapy regimens and number of cycles), hematological and extra-hematological side effects, disease status after induction treatment and during follow-up, and vital status and date of death. Information on subsequent antineoplastic treatment (such as radiation fields and dose) was collected directly from medical records and/or local database. The administrations of GnRH analogue (Decapeptyl) were also evaluated. Information on subsequent secondary malignancies (such as breast cancer) was collected directly from medical records.
MBVD Treatment Plan
The schedule of upfront HL treatment consisted of six MBVD cycles, i.e., 1-day outpatient i.v. infusions of MyocetTM (non-pegylated liposomal doxorubicin, NPLD) at a dose of 25 mg/m2, plus bleomycin 10 mg/m2, vinblastine 6 mg/m2, and dacarbazine 375 mg/m2 on days 1 and 15 of each cycle every month, for twelve administrations according to the Ann Arbor stages and age as already reported15,16. Thus, planned cumulative dose-intensity of NPLD was 300 mg/m2. 
Irradiation 
For those cases with baseline large nodal masses, defined as systemic adenopathy with the largest diameter >5 cm, consolidation radiotherapy (30 Gy) with a linear accelerator was routinely given on residual bulky area, that is, containing residual nodal masses, i.e., post-chemotherapy nodes of size ≥2.5 cm at computed tomography scans, with Deauville scale 5-point scoring system results ≥3 at 2-deoxy-2[F-18] fluoro-D-glucose positron emission tomography assessments, as already reported22-26. Daily fra ction size was about 1.6 Gy five times a week (for a total of 4 weeks).
We defined chest-radiotherapy when it included treatment with any of the following fields: mantle, mediastinal, hemithorax (or anterior fields on one side of chest), whole-lung irradiation, spinal including posterior thoracic/paravertebral, and abdominal (with extension above diaphragm).
Statistical analysis
Standardized incidence ratios (SIRs) were calculated as the ratio of the observed number of breast cancer (BC) cases in the study cohort to the expected number based on age- and calendar year–specific BC incidence rates in the female Italian general population, according to data from the AIRTUM (Associazione Italiana Registri Tumori) Working Group35. Expected numbers of BC cases were obtained by multiplying population incidence rates by the corresponding number of person-years at risk accrued within the cohort. The SIR was computed as the ratio of observed to expected BC cases. Corresponding 95% confidence intervals (CIs) were calculated assuming a Poisson distribution of observed events.
Absolute excess risk (AER) was defined as the difference between observed and expected BC cases divided by the total number of person-years of follow-up and was expressed as excess cases per 10000 person-years.
Women entered the risk set 5 years after completion of frontline HL treatment and were followed until BC diagnosis, occurrence of another secondary malignancy, HL relapse, death, or last available follow-up, whichever occurred first.
All statistical analyses were performed using R software (version 4.3.1). All tests were two-sided, and P values <0.05 were considered statistically significant.


Supplementary Results
According to the most recent AIRTUM data available for the period 2014–2017, the age-standardized incidence rate of female BC among Italian women aged 18–60 years was approximately 86.5 cases per 100000 person-years. In our cohort, comprising approximately 1000 person-years of follow-up, the expected number of BC cases was estimated at 0.86. No BC events were observed during follow-up. Consequently, the SIR was 0.00 (95% CI, 0.00–4.29), calculated using exact Poisson confidence limits for zero-event data. The AER was −8.7 BC cases per 10000 person-years, reflecting fewer observed events than expected based on population incidence rates.
No BC events were observed across predefined clinical subgroups considered at higher risk for secondary breast malignancies (Supplementary Table 1). These included patients exposed to cumulative NPLD doses >200 mg/m² (n=90), women treated before 30 years of age (n=35), patients receiving chest radiotherapy (n=20), those exposed to pelvic irradiation (n=5). Based on age-standardized incidence rates from the Italian general population, the expected number of BC cases ranged from 0.04 to 0.78 across these subgroups, whereas no observed BC events occurred during a median follow-up of approximately 10 years.


Supplementary Table 1. Approximate expected and observed breast cancer cases across predefined clinical subgroups

Clinical subgroup
Patients, 
n (%)
Median follow-up, years
Expected BC cases*
Observed BC cases
Overall cohort
100 (100)
10
0.86
0
Cumulative NPLD dose >200 mg/m²
90 (90)
10
0.78
0
Cumulative NPLD dose ≤200 mg/m²
10 (10)
10
0.09
0
Age <30 years at HL treatment
35 (35)
10
0.30
0
Age 30–40 years at HL treatment
43 (43)
10
0.37
0
Age >40 years at HL treatment
22 (22)
10
0.19
0
Chest radiotherapy
20 (20)
10
0.17
0
No chest radiotherapy
80 (80)
10
0.69
0
Pelvic radiotherapy
5 (5)
10
0.04
0

Abbreviations: BC, breast cancer; GnRH, gonadotropin-releasing hormone; HL, Hodgkin lymphoma; NPLD, non-pegylated liposomal doxorubicin.

* Estimated expected BC cases were obtained by applying the overall age-standardized breast cancer incidence rate reported by the AIRTUM Working Group (86.5 per 100000 person-years) to the approximate person-years accrued within each subgroup. These estimates are descriptive only and were not derived from subgroup-specific population incidence rates.
